# The mediating role of life satisfaction in the relationship between psychological resilience and internet addiction

**DOI:** 10.1038/s41598-026-50933-0

**Published:** 2026-05-07

**Authors:** Nuri Erdemir

**Affiliations:** Department of Guidance and Counselling Psychology, Faculty of Education, Inonu University, Main Campus, 44280 Malatya, Turkey

**Keywords:** Psychological resilience, Life satisfaction, Internet addiction, Structural equation modeling, University students, Health care, Psychology, Psychology, Risk factors

## Abstract

Psychological resilience (PR) and life satisfaction (LS) are considered protective factors for problematic behaviors, yet their structural relationships with internet addiction (IA) and the potential indirect effect of LS require further clarification. This study tested whether LS shows an indirect effect the relationship between PR and IA among university students. The study was conducted with 715 undergraduate students from Türkiye (mean age = 21.6; 55.7% female). Data were collected using the Brief Resilience Scale (ω = 0.88), the Satisfaction with Life Scale (ω = 0.89), and the Young Internet Addiction Test–Short Form (ω = 0.92). Correlation analyses and structural equation modeling (SEM) were performed, and mediation was tested via bootstrap analysis (5,000 resamples). PR was positively related to LS (*r* = .309, *p* < .01) and negatively related to IA, while LS was also negatively related to IA (*r* = –.293 and *r* = –.176, respectively, *p* < .01). The SEM demonstrated good model fit (χ²= 642.35, df = 226, *p* < .001; CFI = 0.941, TLI = 0.935, RMSEA = 0.061, SRMR = 0.042). Standardized path coefficients were PR → LS (β = 0.52, *p* < .01), PR → IA (β = –0.47, *p* < .01), and LS → IA (β = –0.28, *p* < .05). Bootstrap results confirmed that a significant indirect effect was observed the PR–IA association (indirect effect β = –0.15, 95% CI [–0.24, –0.08]). Higher psychological resilience was associated with lower internet addiction both directly and indirectly through greater life satisfaction, indicating that PR and LS function as protective factors against IA in young people. University-based interventions that enhance resilience and life satisfaction may inform prevention efforts targeting problematic internet use.

## Introduction

In today’s society, the widespread use of the internet in daily life offers significant opportunities in terms of communication, access to information, and social interaction^1^ while also increasing concerns about “internet addiction” caused by excessive and uncontrolled use^2^. Although internet addiction has not yet been officially classified as a psychopathological disorder, it is regarded as a major problem that impairs individuals’ capacity to control their online behavior, leading to disruptions in relational, academic, and occupational functioning, and exhibiting characteristics similar to other types of addiction^3,4^. At the same time, the construct remains conceptually and diagnostically contested. Unlike substance-use disorders, a broad ‘internet addiction’ diagnosis has not been formally recognized in major nosological systems, reflecting ongoing concerns about heterogeneity in problematic online behaviors, potential overpathologizing of high engagement, and the difficulty of defining disorder boundaries across diverse internet activities^5^. Notably, the DSM-5 introduced Internet Gaming Disorder only as a condition for further study rather than a formal diagnosis, indicating that evidence was considered insufficient for a generalized internet addiction category^6,7^. Similarly, the ICD-11 includes Gaming Disorder, focusing on persistent impairment and loss of control in gaming specifically, rather than endorsing a unitary ‘internet addiction’ construct^8^. Therefore, in the present study we use the term ‘internet addiction’ in line with the measurement instrument (Young’s Internet Addiction Test–Short Form) and the established survey literature, while acknowledging that our operationalization primarily captures problematic internet use / generalized problematic internet use rather than a universally agreed psychiatric diagnosis. Problematic internet use is often described as a multidimensional phenomenon that can manifest in different activity domains (e.g., gaming, social networking, cybersex, information seeking), which partly explains why a single, universally accepted diagnostic category remains debated. Particularly among the young population, social media platforms, online games, and virtual relationships strengthen compulsive use tendencies, and are associated with higher risk of problematic use^9^.The rise in the popularity of the internet elevates the likelihood of individuals becoming excessively dependent on online platforms, leading to adverse effects on overall well-being^10^. Psychological resilience is defined as the individual’s capacity to adapt and maintain functionality in the face of difficulties, traumas, threats, or significant sources of stress^11^. Resilient individuals possess more functional coping strategies that enable them to manage stressors and preserve emotional balance. In the digital age, psychological resilience is regarded as an important psychological resource that protects individuals from excessively turning to the internet as a means of coping with stress^12^. In contrast, low psychological resilience may render individuals more vulnerable to maladaptive coping strategies—for instance, intense and uncontrolled internet use. We prioritize psychological resilience as the central construct in the model because it represents a broad, higher-order psychological resource reflecting individuals’ capacity to maintain functioning under stress—thereby encompassing adaptive self-regulation and coping flexibility at a more general level. Constructs such as emotion regulation, coping styles, and self-control are closely related but typically specify narrower mechanisms (e.g. regulation strategies, preferred coping patterns, or impulse control) rather than overall adaptive capacity under adversity^13,14^. Accordingly, to reduce construct redundancy and improve theoretical parsimony, we treat resilience as the overarching resource and interpret internet addiction risk in terms of depleted vs. preserved adaptive capacity, while acknowledging that future models could test these more proximal mechanisms explicitly.

Life satisfaction is conceptualized as a subjective indicator of the satisfaction individuals derive from various domains of life through their overall evaluation of their existence and is closely associated with both psychological resilience and internet addiction^15^. It has been demonstrated that individuals with high life satisfaction cope more effectively with stressors and exhibit stronger psychological resilience^16^. Conversely, individuals experiencing low life satisfaction may use the internet more intensely and dysfunctionally to compensate for perceived deficiencies in their offline lives, escape negative emotions, or experience a sense of accomplishment, thereby increasing the risk of internet addiction^17,18^. The compensatory internet use approach emphasizes that elements perceived as missing in life are attempted to be replaced through the internet, and this may particularly may be linked to problematic use in individuals with low life satisfaction^19,20^. The relationships among psychological resilience, life satisfaction, and internet addiction can be conceptualized within the stress and coping framework. According to this model, intense or chronic stress may be associated with lower life satisfaction; the decline in life satisfaction, in turn, may be related to a greater tendency to use the internet as an escape or compensatory tool. On the other hand, high psychological resilience and life satisfaction enable the individual to cope with stress in a more adaptive manner, thus reducing the likelihood of excessive and dysfunctional use of the internet as a coping mechanism. Within this framework, it is considered that psychological resilience and life satisfaction may exert both direct and indirect effects on internet addiction, and that life satisfaction, in particular, may play a mediating role in the relationship between psychological resilience and internet addiction. In this framework, resilience is treated as a broad (distal) resource shaping vulnerability to maladaptive coping (e.g. excessive internet use), while more specific self-regulatory constructs (emotion regulation, coping styles, self-control) are beyond the scope of the present model. Furthermore, cultural values and contextual conditions may shape both stress–coping processes and the forms of problematic internet use; therefore, findings from different cultural contexts can refine theory^21^. In Türkiye, where internet use is widespread, and where relatedness-oriented social expectations can be salient, online environments may be particularly attractive for coping and compensatory motives, making it important to test the model in this context^22,23^. This study aims to fill some of the gaps in the literature by examining the structural relationships among psychological resilience, life satisfaction, and internet addiction in a sample of university students using structural equation modeling (SEM). Structural equation modeling is a powerful statistical approach that combines factor analysis and path analysis, enabling the simultaneous testing of multiple relationships between observed and latent variables^24,25^. This method allows for the testing of theoretically specified complex models, thereby providing a more holistic evaluation of the direct and indirect paths among psychological resilience, life satisfaction, and internet addiction^26,27^. In this context, the present study seeks to elucidate in greater detail the roles of psychological resilience and life satisfaction in the development and maintenance of internet addiction. It is anticipated that the findings will inform prevention-oriented efforts and guide future intervention research aimed at strengthening psychological resilience, enhancing life satisfaction, and reducing the risk of internet addiction^28^.

The study aims to examine the relationships between Psychological Resilience (PR), Life Satisfaction (LS), and Internet Addiction (IA) among university students and to test the mediating role of Life Satisfaction (LS) in the relationship between Psychological Resilience (PR) and Internet Addiction (IA). We hypothesize:

- **H1**: Psychological resilience is positively and significantly associated with life satisfaction.
- **H2**: Psychological resilience is negatively and significantly associated with internet addiction.
- **H3**: Life satisfaction is negatively and significantly associated with internet addiction.
- **H4**: Life satisfaction shows a significant indirect effect in the association between psychological resilience and internet addiction.

## Method

This study employed a cross-sectional design and used structural equation modeling (SEM) to examine the structural relationships among psychological resilience, life satisfaction, and internet addiction. The theoretical model of the research was constructed based on studies in the literature, and the structural relationships among the variables were tested using structural equation modeling^29,30^.

### Participants

The sample of this study consists of a total of 715 undergraduate students enrolled in various faculties of a university in Türkiye. Participants were recruited using a convenience sampling strategy. The sample size was determined using two criteria. First, according to the ‘rule of 10’ in SEM, which suggests at least 10 participants per observed variable^30^, a minimum of 230 participants was required for the 23 items used in this study. Our sample of 715 significantly exceeds this requirement. Second, a post-hoc power analysis was conducted using G*Power 3.1. For a structural model with three latent variables and 715 participants, at an alpha level of 0.05 and a medium effect size (f2 = 0.15), the statistical power (1- β) was calculated as 0.99, which is well above the conventional threshold of 0.80^31^. Of the participants, 398 (55.7%) are female and 317 (44.3%) are male. The distribution by grade level is as follows: 1st year: 144 students (20.1%), 2nd year: 213 students (29.8%), 3rd year: 182 students (25.5%), 4th year: 141 students (19.7%), 5th year: 17 students (2.4%), and 6th year: 18 students (2.5%). The sample comprises students from different faculties and departments, exhibiting a heterogeneous distribution. Although students were drawn from diverse faculties/departments, the sample was recruited from a single university; therefore, generalizability to all university students in Türkiye should be interpreted with caution^32^. The ages of the participants in the study ranged from 17 to 33 years. The mean age of the sample was 21.6 with a standard deviation of 1.86 (SD = 1.86). (Min = 17, Max = 33). The mean age of the participants is 21.6. Prior to data collection, approval was obtained from the relevant university ethics committee, and all participants completed an informed consent form. Participants were informed that the data would be used solely for scientific purposes, and the principle of anonymity was upheld. To reduce social desirability and evaluation apprehension, participants were reminded that there were no right or wrong answers and that responses would be analyzed only in aggregate.

### Data collection tools

All instruments were administered in Turkish, using validated Turkish adaptations of the original scales.

### Brief psychological resilience scale

The scale was developed by Smith et al. (2008)^33^ to measure individuals’ psychological resilience. The Brief Resilience Scale (BRS) is a 6-item, self-report measure with a 5-point Likert-type response format. After reverse-scoring the negatively worded items, higher scores indicate higher psychological resilience. The scale was adapted to Turkish by Doğan (2015)^34^. Sample items include “I tend to bounce back quickly after hard times” (Item 3) and “I usually come through difficult times with little trouble” (Item 5). The confirmatory factor analysis conducted in the present study indicated good model fit (CFI = 0.924, TLI = 0.907, RMSEA = 0.048 [90% CI], SRMR = 0.046). The scale demonstrated high internal consistency, with a McDonald’s Omega (ω) coefficient of 0.88.

### Satisfaction with life scale

The Satisfaction with Life Scale was developed by Diener, Emmons, Larsen, and Griffin (1985)^35^. It is a 5-item scale with a 5-point Likert-type response format that measures individuals’ overall life satisfaction and is widely used to assess subjective well-being. The scale was adapted to Turkish by Dağlı & Baysal (2016)^36^. Sample items include “I am satisfied with my life” (Item 3) and “If I could live my life over, I would change almost nothing” (Item 5). The confirmatory factor analysis conducted in the present study indicated good model fit (CFI = 0.957, TLI = 0.944, RMSEA = 0.045 [90% CI], SRMR = 0.038). The scale demonstrated high internal consistency, with a McDonald’s Omega (ω) coefficient of 0.89.

### Young internet addiction test – short form

The Young Internet Addiction Test – Short Form (IAT-SF), originally developed by Young (1998)^37^ and shortened by Pawlikowski et al. (2013)^38^, consists of 12 items and is a 5-point Likert-type scale. Higher scores on the scale indicate higher levels of internet addiction. The scale was adapted to Turkish by Kutlu, Savcı, Demir & Aysan (2016)^39^. Sample items include “How often do you stay online longer than you intended?” (Item 1) and “How often do you lose sleep due to late-night internet use?” (Item 6). The confirmatory factor analysis conducted in the present study indicated good model fit (CFI = 0.945, TLI = 0.932, RMSEA = 0.051 [90% CI], SRMR = 0.042). The scale demonstrated high internal consistency, with a McDonald’s Omega (ω) coefficient of 0.92. Consistent with the ongoing nosological debate, IAT-SF scores in this study are interpreted as an index of problematic internet use severity rather than a categorical psychiatric diagnosis.

### Data analysis

The data analysis process was conducted in several stages:

1. Preliminary Analyses: The data were transferred to SPSS v22 and screened for missingness; no missing data were detected, so no missing-data procedure was applied. Subsequently, outliers were checked using the Mahalanobis distance, and observations exceeding the critical value of 49.73 (χ²^23^, *p* < .001) were excluded^40^.
2. Normality Test: Skewness and kurtosis values of the variables were examined, and all variables were found to fall within the ± 2 range. This result indicates that the normality assumption was met^41^. Additionally, the Kolmogorov-Smirnov and Shapiro-Wilk tests provided findings supporting normality. In addition to evaluating univariate normality through skewness and kurtosis values, the assumption of multivariate normality was assessed using Mardia’s coefficient. Given that Structural Equation Modeling (SEM) with Maximum Likelihood estimation is sensitive to multivariate non-normality, this step is crucial for the stability of the parameter estimates^30^. Analysis of the data (*N* = 715) revealed that Mardia’s multivariate kurtosis coefficient was 14.28 with a critical ratio (C.R.) of 3.45. Since the critical ratio was below the threshold of 5.0, the data were considered to meet the assumption of multivariate normality sufficiently, justifying the use of Maximum Likelihood (ML) estimation for the structural model.
3. Multicollinearity: Prior to structural equation modeling, multicollinearity among the variables was examined. VIF values ranged between 1.50 and 1.90, and tolerance values were above 0.52. These values indicate that there is no multicollinearity problem^30^.
4. Correlation Analysis: Relationships among the variables were tested using Pearson correlation coefficients. This step allowed the determination of the direction of relationships among variables before proceeding to the structural model.
5. Structural Equation Modeling: AMOS v24 software was used in the analysis process. Direct effects, indirect effects, and total effects were calculated separately to test the validity of the model. SEM analysis is based on linear regression-based path analysis specifically, the Maximum Likelihood (ML) estimation method was employed to estimate the model parameters^42^. Internal consistency was evaluated using McDonald’s Omega (ω) instead of Cronbach’s Alpha, as Omega does not require the assumption of tau-equivalence and provides a more robust estimate of reliability in latent variable modeling^43^.
6. Model Fit Indices: Model fit was evaluated using several goodness-of-fit indices. Following the recommendations of^30^, the normed chi-square ( χ²/df) was not utilized as a primary fit index, as it is based on a flawed logic of correcting for sample size. Instead, given our large sample size (*N* = 715), we focused on alternative fit indices that are less sensitive to sample size but robust in assessing model-data discrepancies, specifically the Comparative Fit Index (CFI), Tucker-Lewis Index (TLI), Root Mean Square Error of Approximation (RMSEA), and Standardized Root Mean Square Residual (SRMR). Values of CFI and TLI of 0.90 or higher, together with RMSEA and SRMR values below 0.08, were considered indicative of acceptable model fit^44,45^.
7. Bootstrap Analysis: The bootstrap method was applied to test whether life satisfaction plays a mediating role in the relationship between psychological resilience and internet addiction in the structural model. Bootstrap resampling was performed with 5,000 samples, and the significance of indirect effects was examined at the 95% confidence interval^46,47^. A confidence interval that does not include zero indicates that the mediation effect is statistically significant.

## Results

The descriptive statistics for the variables of psychological resilience (PR), life satisfaction (LS), and internet addiction (IA) included in the study are presented in Table 1. The mean psychological resilience score of the participants was 3.16, indicating that participants generally possess moderate-to-high levels of psychological resilience. The mean life satisfaction score was 3.00, revealing that the sample exhibits moderate life satisfaction. The mean internet addiction score was found to be 2.50; this value indicates that participants’ internet addiction tendencies are at a low-to-moderate level.

**Table 1:** Descriptive Statistics (*N* = 715).

Variable	Mean	SD	Min	Max
Psychological Resilience	3.16	0.65	1.00	5.00
Life Satisfaction	3.00	0.70	1.00	5.00
Internet Addiction	2.50	0.67	1.08	5.00

N = sample size; SD = standard deviation; Min = minimum; Max = maximum.

### Correlation analysis

The relationships among the variables were examined using Pearson correlation coefficients, and the results are presented in Table 2.

**Table 2:** Correlations Among Variables (*N* = 715).

Variable	PR	LS	IA
Psychological Resilience (PR)	1	0.309**	–0.293**
Life Satisfaction (LS)	0.309**	1	–0.176**
Internet Addiction (IA)	–0.293**	–0.176**	1

Correlations significant at *p* < .01 are indicated with **.

A positive and significant relationship was found between psychological resilience and life satisfaction (*r* = .309, *p* < .01). This finding suggests that individuals with higher psychological resilience may also have higher life satisfaction^48^. A negative and significant relationship was identified between psychological resilience and internet addiction (*r* = –.293, *p* < .01). This result indicates that individuals with higher psychological resilience are less likely to engage in problematic internet use as a way of coping with stress^37^. The relationship between life satisfaction and internet addiction was weak but significantly negative (*r* = –.176, *p* < .01). This finding demonstrates that as life satisfaction increases, the tendency toward internet addiction decreases. The relationship between life satisfaction and internet addiction was weak but significantly negative (*r* = –.176, *p* < .01), suggesting a small association.

### Measurement model and validity

Before testing the hypothesized structural relationships, a full measurement model (overall CFA) was tested to ensure that the latent variables were distinct and that the observed items accurately represented their respective constructs. The measurement model included Psychological Resilience (PR), Life Satisfaction (LS), and Internet Addiction (IA) as correlated latent factors.

The results of the confirmatory factor analysis (CFA) indicated that the measurement model fit the data well. In accordance with the recommendations of^30^, the χ 2 /df ratio was not used to evaluate model fit due to its sensitivity to sample size and flawed logic in correcting for degrees of freedom. Instead, alternative fit indices were prioritized. The model demonstrated a robust fit to the data: χ² = 642.35, df = 226, *p* < .001, CFI = 0.941, TLI = 0.935, RMSEA = 0.061 (90% CI: 0.056 − 0.066), and SRMR = 0.042. Although the Chi-square test was statistically significant, this was expected given the large sample size (*N* = 715). All standardized factor loadings were statistically significant (*p* < .001) and ranged from 0.62 to 0.88, exceeding the recommended threshold of 0.50. These results confirm that the observed variables adequately represent their respective latent constructs, providing a solid foundation for the subsequent structural model analysis. To evaluate the internal consistency and convergent validity, McDonald’s Omega (ω), Composite Reliability (CR), and Average Variance Extracted (AVE) were calculated (Table 3). The CR values for all constructs were above 0.70, and the AVE values exceeded the recommended level of 0.50, indicating strong convergent validity.

**Table 3:** Reliability and Convergent Validity of the Measurement Model.

Construct	Items	McDonald’s ω	CR	AVE
Psychological Resilience (PR)	6	**0.88**	0.884	0.561
Life Satisfaction (LS)	5	**0.89**	0.912	0.675
Internet Addiction (IA)	12	**0.92**	0.935	0.543

PR = Psychological Resilience; LS = Life Satisfaction; IA = Internet Addiction; ω = McDonald’s omega; CR = composite reliability; AVE = average variance extracted.

Discriminant validity was assessed using the Fornell-Larcker criterion. As shown in Table 4, the square root of the AVE for each latent variable (shown in bold on the diagonal) was higher than its correlation with any other construct, confirming that the constructs are statistically distinct.

**Table 4:** Discriminant Validity Results (Fornell-Larcker Criterion).

Construct	1 (PR)	2 (LS)	3 (IA)
1. Psychological Resilience (PR)	**(0.749)**		
2. Life Satisfaction (LS)	0.309**	**(0.821)**	
3. Internet Addiction (IA)	− 0.293**	− 0.176**	**(0.737)**

PR = Psychological Resilience; LS = Life Satisfaction; IA = Internet Addiction; AVE = average variance extracted. Diagonal values in parentheses represent the square root of the AVE. ** indicates *p* < .01.

### Testing of the structural model

**Fig. 1:**
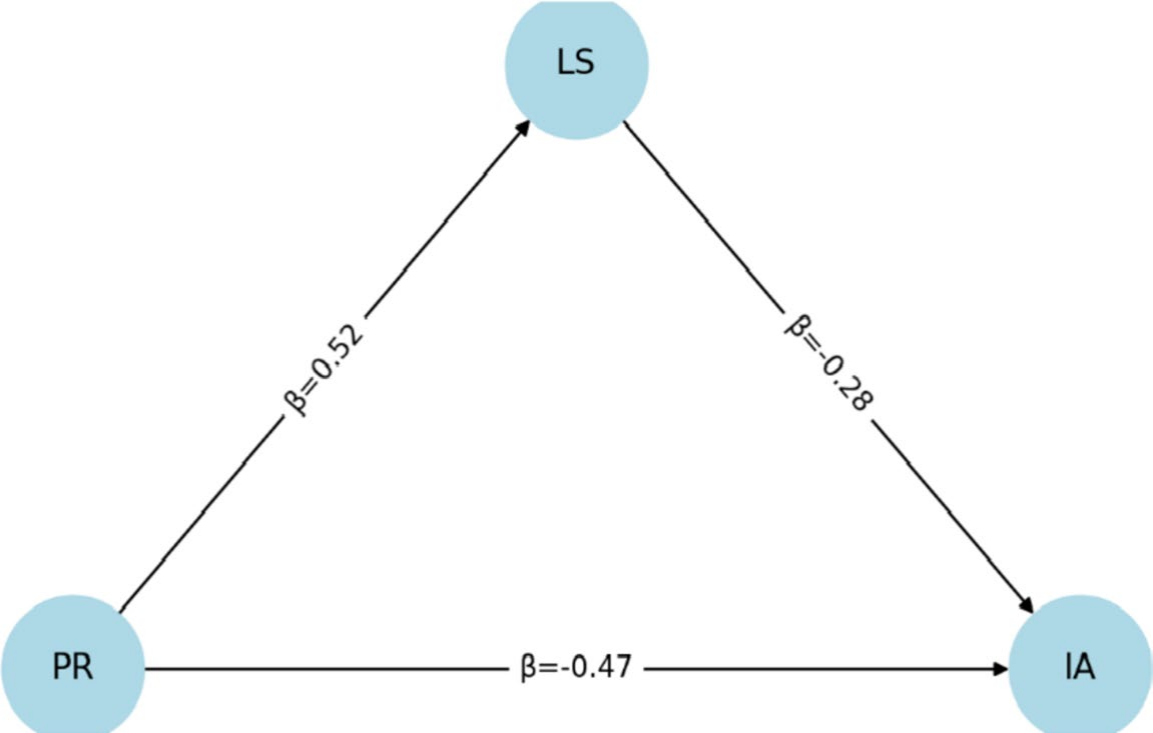
Path diagram for psychological resilience (PR), life satisfaction (LS), and internet addiction (IA).

The structural equation model proposed in the study was tested, and the model fit indices were calculated. The structural relationships among psychological resilience, life satisfaction, and internet addiction are presented in Fig. 1.

Preliminary analyses indicated that the data set satisfied the requirement for multivariate normality (Mardia’s C.R. = 3.45 < 5.0). Consequently, the structural model was tested using the Maximum Likelihood method. The structural model results were as follows: χ²= 642.35, df = 226, *p* < .001. While the Chi-square test was statistically significant, this was expected due to the large sample size, which increases the power of the test to detect even minor discrepancies. Therefore, the overall model fit was interpreted through alternative indices: CFI = 0.94, TLI = 0.93, RMSEA = 0.06, and SRMR = 0.04. These values indicate an acceptable and robust fit between the hypothesized model and the observed data^42,44^.

### Testing of hypotheses

H1: Psychological resilience was positively and significantly associated with life satisfaction (β = 0.52, *p* < .01); therefore, H1 was supported. This finding indicates that resilient individuals experience higher life satisfaction due to their more effective coping processes with difficulties^48,49^.

H2: Psychological resilience was negatively and significantly associated with internet addiction (β = –0.47, *p* < .01); therefore, H2 was supported. The possession of more functional coping strategies by individuals with high psychological resilience can be considered a factor that prevents them from using the internet at an addictive level^37^.

H3: Life satisfaction was negatively and significantly associated with internet addiction (β = –0.28, *p* < .05); therefore, H3 was supported. Higher life satisfaction reduces individuals’ need for satisfaction derived from the online world, thereby lowering the risk of addiction.

H4: Life satisfaction indirect effect the relationship between psychological resilience and internet addiction. In the bootstrap analysis, the indirect effect of the path psychological resilience → life satisfaction → internet addiction was found to be significant (Bootstrap β = –0.15, 95% CI [–0.24, –0.08]). The fact that the confidence interval does not include zero indicates that the indirect effect is significant^46,47^. Therefore, H4 was accepted.

The findings indicate that psychological resilience is associated with higher life satisfaction and lower internet addiction, whereas life satisfaction is associated with lower internet addiction; together, these variables may function as protective correlates. The findings are also consistent with previous studies in the literature^37,48,49^.

## Discussion and conclusion

In this study, the relationships among psychological resilience, life satisfaction, and internet addiction were examined using structural equation modeling. The analysis findings revealed that all four theoretically proposed hypotheses were supported. Psychological resilience was found to significantly and positively associated with life satisfaction and negatively associated with internet addiction; life satisfaction was negatively associated with internet addiction; furthermore, life satisfaction was found to play a partial mediating role in the relationship between psychological resilience and internet addiction.

The first finding is that psychological resilience was positively associated withlife satisfaction (β = 0.52, *p* < .01). This result indicates that individuals with higher levels of psychological resilience derive greater satisfaction from their lives. Similar results exist in the literature. For instance, Connor and Davidson (2003)^49^ stated that individuals with high psychological resilience cope more effectively with stress and therefore exhibit higher subjective well-being. Ryff and Singer (2003)^48^ also argued that life satisfaction is one of the strongest outcomes of psychological resilience. This finding reaffirms that psychological resilience is a critical determinant of subjective well-being.

The second finding is that psychological resilience was negatively associated with internet addiction (β = –0.47, *p* < .01). This result shows that individuals with higher psychological resilience are less likely to use the internet at an addictive level. Young (1998)^37^ suggested that individuals who are inadequate in coping with stress are at greater risk for internet addiction. Accordingly, the findings obtained in this study support Young’s model and demonstrate that psychological resilience serves a protective function against internet addiction.

The third finding is that life satisfaction was negatively associated with internet addiction (β = –0.28, *p* < .05); however, the magnitude of this association was modest. Higher life satisfaction may reflect greater offline fulfillment and psychosocial adjustment, which can be linked to a lower tendency to use the internet excessively for compensation. Diener et al. (1985)^35^ emphasized that life satisfaction plays a central role in individuals’ psychosocial adjustment. Consistent with this view, our results suggest that life satisfaction is a complementary correlate of internet addiction rather than a stand-alone lever for reducing problematic internet use. The fourth finding reveals that life satisfaction indirect effect the relationship between psychological resilience and internet addiction (Bootstrap β = –0.15, 95% CI [–0.24, –0.08]). Hayes (2009)^46^ noted that indirect effects in mediation analyses play a critical role in explaining how an independent variable influences a dependent variable. In this study, psychological resilience was linked to lower internet addiction through higher life satisfaction (i.e. a significant indirect effect). This result indicates that psychological resilience and life satisfaction should be considered together when explaining internet addiction. In interpreting this indirect effect in the Turkish sample, sociocultural factors may also be relevant. In Türkiye, life satisfaction may be shaped not only by individual achievement or personal well-being, but also by family relationships, interpersonal connectedness, and culturally salient social expectations. In such a context, students with higher psychological resilience may be better able to manage relational and social pressures, maintain more satisfying family and peer relationships, and preserve a more positive evaluation of their lives. In turn, greater life satisfaction may reduce the tendency to use the internet excessively for escape, compensation, or emotional relief. Therefore, the mediating role of life satisfaction in this study may be understood not only as an individual psychological process but also as one that is embedded in the broader sociocultural context of the Turkish university student experience.

### Theoretical contributions

This research contributes to the literature in three ways. First, it confirms the validity of previous international findings^48,49^ by testing the relationship between psychological resilience and life satisfaction in a Turkish sample, where sociocultural emphases on relatedness may shape stress appraisals and coping preferences^23^. Second, it demonstrates that psychological resilience not only increases life satisfaction but also exerts a strong negative effect on internet addiction. Third, the mediating role of life satisfaction sheds light on a relationship that has been insufficiently studied in the literature and suggests a potential conceptual framework for understanding the association between psychological resilience and internet addiction, which should be further examined in longitudinal and experimental research.

### Practical contributions

The findings may inform prevention-oriented practices for educators and psychological counselors; however, given the cross-sectional and correlational design, they should not be interpreted as evidence for intervention efficacy.

- Universities may consider offering programs that support psychological resilience (e.g., stress coping, problem-solving, resilience training).
- Psychosocial supports that may enhance students’ life satisfaction can be considered as complementary components of broader prevention efforts.
- Digital literacy and leisure-time management initiatives may be considered to support healthier coping and reduce problematic internet use.

These suggestions are offered as practice-relevant implications that require evaluation in longitudinal and intervention studies.

### Limitations and future research

Although the findings of this study provide significant contributions, they are subject to certain limitations.

- The sample consists solely of university students; therefore, the generalizability of the findings to different age groups and cultural contexts is limited. Future research is recommended to include samples from diverse age groups and to examine whether these associations vary across cultural contexts.
- The data were collected using self-report measures, which may introduce the risk of social desirability bias. Future studies could employ multiple data collection methods (e.g. observation, qualitative interviews).
- The study is based on a cross-sectional design; therefore, definitive causal inferences cannot be made. Longitudinal studies could reveal the developmental trajectory of the relationships among psychological resilience, life satisfaction, and internet addiction over time. Therefore, the estimated indirect effect should be interpreted as a statistical association rather than evidence of temporal or causal mediation^50^.
- Conceptual and diagnostic debates remain regarding a unitary ‘internet addiction’ construct; therefore, our findings should be interpreted in relation to self-reported problematic internet use as operationalized by the IAT-SF, rather than as evidence about a formally established psychiatric disorder.
- Related constructs (emotion regulation, coping styles, self-control) overlap conceptually with resilience; future studies should test whether resilience remains associated with problematic internet use when these more proximal mechanisms are modeled simultaneously.

All variables were assessed via self-report, which may introduce social desirability and common method variance; future studies should incorporate multi-method or multi-informant data (e.g., behavioral logs, peer/parent reports)^51^.

This research has revealed that psychological resilience and life satisfaction exert both direct and indirect effects on internet addiction. It has been determined that individuals with higher levels of psychological resilience also exhibit higher life satisfaction, and this situation reduces internet addiction. The findings demonstrate that psychological resilience and life satisfaction serve as protective factors against internet addiction among young individuals. In conclusion, it can be stated that psychoeducational and social support programs implemented in universities may support prevention efforts by strengthening students’ psychological resilience and, to a lesser extent, life satisfaction.

## Data Availability

The dataset generated and/or analyzed during the current study is publicly available on https://zenodo.org/records/18601476.
